# A psychosocial bouldering intervention improves the well-being of young refugees and adolescents from the host community in Lebanon: results from a pragmatic controlled trial

**DOI:** 10.1186/s13031-024-00615-3

**Published:** 2024-09-14

**Authors:** Katharina Luttenberger, Beat Baggenstos, Charbel Najem, Charles Sifri, Piotr Lewczuk, Anne Radegast, Simon Rosenbaum

**Affiliations:** 1 Department Medical Psychology and Medical Sociology, Faculty of Medicine, Friedrich-Alexander-Universität Erlangen-Nürnberg, Erlangen, Germany; 2 ClimbAID, Zurich, Switzerland; 3 Spine, Head and Pain Research Unit Ghent, Department of Rehabilitation Sciences, Faculty of Medicine and Health Sciences, Ghent University, Ghent, Belgium; 4 Department of Physiotherapy, Faculty of Public Health, Antonine University, Baabda, Lebanon; 5 Branch of ClimbAID, ClimbAID Lebanon, Zurich, Switzerland; 6 Department Psychiatry and Psychotherapy, Faculty of Medicine, Friedrich-Alexander-Universität Erlangen-Nürnberg, Erlangen, Germany; 7 Department of Neurodegeneration Diagnostics, Medical University of Białystok, Białystok, Poland; 8 Department of Biochemical Diagnostics, University Hospital of Białystok, Białystok, Poland; 9 School of Clinical Medicine, Discipline of Psychiatry and Mental Health, Faculty of Medicine and Health, UNSW, Sydney, Australia

**Keywords:** Lebanon, Forced displacement, Syria, Refugee, Physical activity, MHPSS, Host community, Bouldering, Climbing therapy

## Abstract

**Background:**

Mental health and psychosocial support (MHPSS) is increasingly considered vital for addressing the needs of displaced communities. The mental health of young people in Lebanon, including members of the host community and refugees, has been severely affected by multiple crises. Physical activity (PA) is an effective means for enhancing mental health, but evidence of PA’s impact on mental health among forcibly displaced populations is still emerging and often varies widely across studies.

**Method:**

In this waitlist-controlled study, we examined the effectiveness of an 8-week psychosocial bouldering group intervention offered by the nonprofit organization ClimbAID on psychological well-being, distress, self-efficacy, and social cohesion in a group of mostly Syrian refugee adolescents residing in the Bekaa Valley, Lebanon. The intervention consisted of 8 sessions and took place once a week for 2 h in a group of up to 12 adolescents with 2 trained facilitators and up to 2 volunteers, supervised by a climbing instructor and a social worker. Multilevel analyses were performed for all outcomes.

**Results:**

233 people were included in the study. The dropout rate was approximately 33%. The IG improved significantly more than the waitlist group in terms of overall mental well-being and psychological distress. Group allocation was a significant predictor of improvements in mental well-being and psychological distress and showed a trend toward predicting self-efficacy. There was no positive impact of the intervention on social cohesion.

**Conclusions:**

Even in complex humanitarian settings of forced displacement, a psychosocial bouldering intervention reduces psychological distress and increases well-being in a mixed group of host and refugee youth in Bekaa, Lebanon.

**Trial registration:**

Prospectively registered with ISRCTN 13005983, registered April 1st, 2022.

**Supplementary Information:**

The online version contains supplementary material available at 10.1186/s13031-024-00615-3.

## Background

In the context of forced displacement, children and adolescents are one of the groups most at risk in terms of physical, social, and mental well-being [1]. In 2023, they accounted for 40% of all refugees [2]. Providing mental health and psychosocial support (MHPSS), defined by the Interagency Standing Committee (IASC) Reference Group on MHPSS in Emergency Settings as “any type of local or outside support that aims to protect or promote psychosocial well-being and/or prevent or treat mental disorder” (p. 4), is increasingly recognized as a critical component of a holistic response to addressing the needs of forcibly displaced communities [3, 4]. Recent meta-analyses [5, 6] on MHPSS interventions with adolescents in low- and middle-income countries (LMICs) showed positive outcomes, especially regarding posttraumatic stress disorder (PTSD) and depression symptoms but also the risk for unintended outcomes [5]. Most studies were conducted in schools or at the family level and focused on treating rather than preventing mental health conditions [7]. Whereas most refugees reside in LMICs, most research on MHPSS interventions has been conducted in high-income countries [8]. In addition, more than half of the studies have relied on pre-post designs without a control group, with control-group studies always having smaller effect sizes [9].

One intervention that can be implemented within both clinical and social-environmental frameworks [10] is physical activity (PA). PA—defined as “*people moving, acting, and performing within culturally specific spaces and contexts*” [11]—is an efficacious strategy for protecting and promoting mental health [12, 13], as either a stand-alone or adjunct strategy [14]. Numerous meta-analyses [14–16] and Mendelian randomization studies have shown causal relationships between PA and the reduction of depression [5, 17, 18]. One specific type of PA shown to be efficacious in improving mental health outcomes is a modality of climbing called *bouldering*, which is performed without the use of ropes or harnesses [19]. A recent meta-analysis showed that therapeutic climbing/bouldering has positive effects on physical, social, and mental well-being [20]. For example, in a randomized controlled trial involving *N* = 156 German outpatients experiencing a depressive episode, a group psychotherapeutic bouldering intervention was found to be equally as effective as group cognitive behavioral therapy [21] and more effective than PA alone in reducing depressive symptoms and enhancing a feeling of self-efficacy [22].

Findings on PA, mental health, and forced displacement are emerging. For example, a 2021 meta-analysis of 27 studies that included refugees and asylum-seekers reported that PA effectively decreases mental health problems and promotes overall functioning, self-efficacy, and coping resources [23]. From an implementation perspective, humanitarian organizations, including the United Nations International Agency for Migrations (IOM) [24] and the International Federation Red Cross (among others), offer documents that provide guidance on the intersection of sports and MHPSS [25]. Studies on PA in the context of forced displacement often rely on pre-post analyses and show high levels of heterogeneity. Thus, the optimal approach for promoting PA in these contexts remains uncertain [23].

The small, middle east country of Lebanon is host to one of the largest numbers of refugees per capita compared with any other country. This count includes an estimated 1.5 million displaced Syrians [26], about 250,000 Palestinian refugees [27], and more than 13,500 refugees of other nationalities. This influx of refugees is a challenge [28] for a country dealing with one of the most devastating and prolonged economic crises [29] that was further compounded by both the COVID-19 pandemic and the Beirut port explosion in 2020 [30]. A 2020 study conducted on 108 Syrian refugees in the Bekaa region, located approximately 30 km East of the capital Beirut near the Syrian border, found that 74% experienced at least one symptom of PTSD, and among those under 30 years of age, 84% believed they had no future for themselves or their families [31, 32]. A subsequent meta-analysis found that the prevalence of PTSD, depression, and anxiety in displaced Syrians was up to 7–8 times higher than in the general population [33]. In a recent study [34] on mental health in minor refugees in Lebanon, more than half of all children and adolescents met the criteria for at least one psychiatric diagnosis with high rates of PTSD (39.6%), conduct/oppositional defiant disorder (26.9%), depression (20.1%), and anxiety disorders (47.8%). Exposure to war-related events, maltreatment, and conflict with a caregiver were the traumatic events most often associated with PTSD and depression. Furthermore, the adverse living conditions have placed an additional stress on already overburdened health services [28, 35] and have contributed to a deterioration of the relationship between the Lebanese host community and the Syrian refugee community [36].

There have been numerous calls to strengthen the MHPSS provided to displaced people in Lebanon, despite the already overburdened services [37]. As one of four strategic goals, Lebanon’s national mental health strategy 2023–2030 lists the “increased availability of acceptable, affordable, and accessible comprehensive, integrated, person-centered and responsive mental health services in community-based settings and decrease institution-based services” [38].

The nonprofit organization ClimbAID has offered MHPPS-informed bouldering programs on a community level for different groups of participants of all nationalities in Bekaa since 2017. The YouCLIMB bouldering intervention for adolescents between 14 and 19 integrates climbing therapy and experiential education by drawing on previous studies on bouldering psychotherapy [19, 21, 39]. Rooted in Miller et al.’s [10] clinical framework, ClimbAID’s theory of change assumes positive psychological changes on the levels of prevention and treatment [7] if participants take part in the program’s module topics, including trust and respect, collaboration and teamwork, communication and conflict resolution, and problem solving and decision making. Bouldering itself—as well as specifically designed climbing exercises—mirror the behavioral and thought patterns of the real world and are purposefully worked on in the MHPSS bouldering sessions. It is assumed that insights and experiences in the climbing session can be transferred to day-to-day behavior, thus increasing mental well-being. Moreover, the climbing community fosters a robust culture of inclusivity, respect, self-reflection, and continual learning, offering a framework in which participants can navigate their identities.

We therefor hypothesized that ClimbAID’s psychosocial bouldering intervention YouCLIMB would improve mental well-being compared with a waitlist control group (CG).

Additionally, we evaluated the impact of the intervention on self-efficacy, psychological distress, and social cohesion. The protocol has been published elsewhere [40].

## Methods

### Study design

**Fig. 1:**
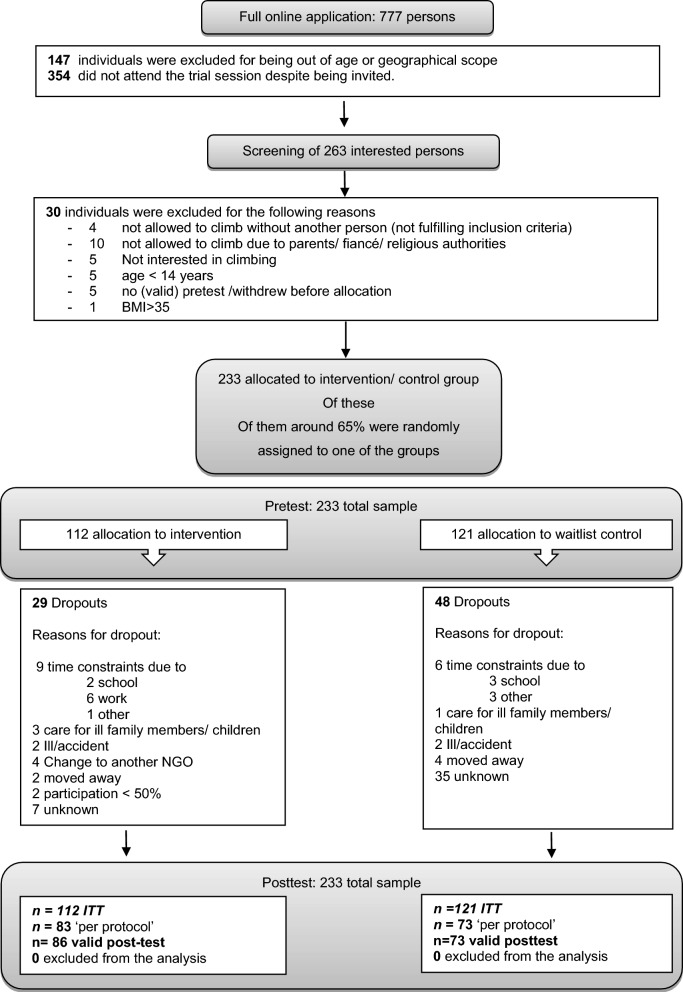
Consort flow chart

In this waitlist control group study, we investigated the effectiveness of a psychosocial bouldering intervention on several mental health outcomes for adolescent refugees and adolescents from the host community in Bekaa, Lebanon. The full study protocol was published elsewhere [40], and the study was prospectively registered with ISRCTN 13005983 in April 2022. Recruitment began in April 2022 and continued until September 2022. The intervention was delivered in three cycles, with each intervention lasting for 8 weeks. The first cycle began in May 2022 and the last one in October 2022. Data collection was finalized in November 2022. Participants were allocated to either the intervention group (IG) or a waitlist control group (CG), who were given the opportunity to participate in the intervention 8 weeks later (see Fig. 1).

### Community involvement and ClimbAID’s activities

Ever since its projects began in 2017, ClimbAID has been continuously active in the Bekaa region of Lebanon and has established a strong presence in the local communities.

Facilitators and beneficiaries from the host and refugee communities as well as local and international nonprofit organizations have been instrumental in the development and implementation of the YouCLIMB intervention, which has continuously evolved over a period of 3 years. To refine the study design, research methods, outreach, and questionnaire analysis and administration, ClimbAID actively collaborated with local team members and previous participants. Important changes to the planned administering of the questionnaires go back to these discussions (see data collection). To construct guidelines for the interviews, in-depth interviews with previous participants and volunteers were conducted. Findings were shared with the local staff and will be made accessible to the community after publication.

### Inclusion and exclusion criteria

We recruited adolescents from both the host community and refugee population residing in the Bekaa valley in Lebanon. In order to meet the requirements, adolescents had to fulfill only a few criteria: (a) their age had to fall within the age range of 14 to 19 years, (b) they had to provide informed consent, and (c) we needed informed consent from their legal guardian. Participants were required to be able to attend the bouldering intervention and participate in data collection. The study area was limited by the towns of Zahle to the north, Deir Zenoun and Marj to the south, and Qab Elias/Bouarej to the west. Exclusion criteria included: (a) pregnancy or contraindications specified by a doctor or (b) a body mass index (BMI) below 18.5 (underweight) or above 35 (obesity Class III). Inclusion and exclusion criteria were mostly assessed via self-report, and BMI was calculated by study personnel on the basis of participants’ self-reported weight and height.

### Enrollment strategies

ClimbAID recruited participants via multiple channels, including visits in informal tented settlements, schools, nonprofit organizations, and ClimbAID social media campaigns. Interested parents or legal guardians could use an online form to apply for their adolescents to participate, and eligible adolescents were invited to a trial session to confirm their interest in participating. Support was offered to interested applicants or their parents who struggled to complete the form by themselves. Written informed consent was obtained from both the adolescents and their legal guardians following communication with trained ClimbAID staff. For a more detailed description, please see [40].

### Group allocation

Allocation to either group was random except in cases where, due to cultural and logistical constraints, it was not feasible to implement a strict randomization procedure. For instance, some potential participants could attend only one of the three intervention cycles due to school obligations, whereas some girls could participate only if accompanied by a family member. To enhance adherence to the intervention, participants were allowed to choose the timing of the intervention cycle (April, June, or September), provided they were available at each measurement point. In about 35% of the cases, this led to nonrandom assignment to the IG or CG. The remaining participants were randomly assigned to either the control or IG by the local program manager of ClimbAID in Lebanon. A random number was generated for each candidate using the = RAND() function in Microsoft Excel, and the parity of the thousandth digit of the random number determined the group assignment. Candidates were assigned to the CG if the thousandth digit was even and to the IG if it was odd.

### Power

The necessary sample size for the study was calculated for the primary outcome Mental Wellbeing using the G*Power software, with a two-tailed alpha of 0.05, a power of 80%, and an estimated unadjusted effect size of Cohen’s *d* = 0.55 [19], which resulted in a required sample size of 52 participants per group. To account for a dropout rate of around 35%, we aimed to enroll 80 participants in each group (i.e., intervention and control), resulting in a total of at least 160 adolescents distributed across six smaller bouldering groups (each consisting of 12–14 participants) and one large CG (see [37]).

### Data collection

Data were collected at two timepoints: before (t0) and after the intervention (t1; before the CG was offered the intervention). Data comprised validated questionnaires on mental well-being, psychological distress, self-efficacy, and social cohesion. Data were collected in groups of up to 14 participants by trained study staff. Most of the study personnel had a similar background (either Syrian or Lebanese) to that of the participants. This similarity helped prevent misunderstandings due to differences in Arabic dialects. All participants were invited to the ClimbAID site at their respective time points where they were given paper questionnaires. All participants were asked to complete the questionnaires themselves. As a certain proportion of Syrian refugee youth experience reading difficulties, which are often not shared freely, the questions were also read aloud, and the response options were explained. The study personnel were trained not to influence the answers [40]. Due to the nature of the intervention and the use of self-rating questionnaires, it was not possible to blind participants and facilitators.

### Adverse events

Facilitators assessed adverse events during the entire intervention phase and again at both timepoints. Mild adverse events consist of various minor superficial injuries (e.g., bruises or scratches) that are transient and do not necessitate treatment. Moderate adverse events include transient injuries requiring medical attention, such as ligament sprains or fractures of the limbs, whereas severe adverse events entail serious head injuries, spinal cord injuries, death, or suicidal attempts. Mild, moderate, and severe adverse events were documented in the IG at each session if they occurred and at both timepoints. The CG was asked to report moderate and severe adverse events at the measurement points. Mild adverse events were not documented for the CG.

Criteria for discontinuation of the trial was if three or more, moderate adverse events occurred in the IG compared to the waitlist group, and these were determined to be a direct result of the intervention. Mild adverse events were recorded but did not lead to the discontinuation of the program. In the event of a severe adverse event directly caused or associated with the intervention, the study would have been immediately discontinued [40].

Additionally, qualitative interviews were conducted with a subgroup of participants during the intervention by trained interviewers in the appropriate Arabic dialect. Interviews took place at the end of the bouldering session in a confidential atmosphere and were conducted by external staff. The results of these qualitative data have yet to be completed and will be published elsewhere. Participation was voluntary, and participants were free to leave the study at any time. All procedures were approved by the Ethics Committee of Antonine University (approval number 279–2022 in February 2022) and conformed to the principles embodied in the Declaration of Helsinki.

### Intervention

The YouCLIMB bouldering intervention combines climbing therapy and experiential education and builds on prior studies on bouldering psychotherapy [19, 21, 39]. ClimbAID’s theory of change uses specifically designed bouldering exercises in different modules (see Table 1) to reflect real-world behaviors and thought patterns that are then individually addressed in reflection groups with the goal of stimulating participants to apply the lessons they learned in climbing to daily life. In addition, sessions are aimed at improving resilience by encouraging participants to overcome obstacles and setbacks on the climbing wall by fostering an atmosphere of learning and a sense of determination and perseverance. The YouCLIMB intervention is the result of a 3-year development process that has incorporated participants’ wishes, topics, and frequently mentioned difficulties. Focusing on module topics and core themes, such as shared leadership, emotional education, and mindfulness, the intervention strives to equip participants with crucial life skills while fostering resilience, self-awareness, and interpersonal relationships within a supportive community. Sessions were held weekly for 2 h at an artificial bouldering wall in Taanayel on the premises of the Lebanese nonprofit organization Arcenciel. Sessions were facilitated by Local ClimbAID team members, trained and supervised by a certified climbing instructor and a social worker with experience conducting interventions with refugee youth. Facilitators underwent training in psychosocial support, child protection, and mental health first aid, alongside intensive instruction in the YouCLIMB curriculum.

**Table 1:** Session-specific topics in the bouldering intervention (see [40])

Session	Module themes	Cross-cutting themes
1	Trust & respect	Shared leadership Emotional education Mindfulness
2		
3	Cooperation & teamwork	
4		
5	Communication & conflict resolution	
6		
7	Problem-solving & decision-making	
8		

Additionally, senior organization members regularly discussed and reviewed all bouldering sessions with the responsible facilitator to promptly address any challenges.

At the beginning of each session, the facilitators welcomed the participants, reminded them of the group’s rules, and introduced the theme and objectives of the current session. The sessions then began with a breathing meditation exercise, followed by a warm-up routine. During the activity part of the session, the participants engaged in experiential bouldering activities and games designed to demonstrate and experience the session’s objectives. For example, Lesson 3 is about teamwork and cooperation. This topic is illustrated by a game called "Three-legged Climbing." In this game, two players are connected by a string at their ankles and have to climb a bouldering wall together. Success requires full concentration, empathy, clear communication, trust and respect—all elements of teamwork and cooperation. During the exercise, participants are encouraged to try different options, for example, to ask for help, to wait for the other person, and to communicate their needs. The participants will then meet in a reflection group to talk about their experiences, share what they have learnt and how they can apply their learning to everyday life. Table 1 presents an overview of the sessions.

YouCLIMB is embedded in other bouldering-related activities by ClimbAID. Graduates of YouCLIMB are offered opportunities to take part in open bouldering sessions, to become part of the ClimbAID climbing team, to volunteer for future YouCLIMB cycles, or to take part in the ClimbABILITY program, which is aimed at children and young people with disabilities. In addition, outdoor climbing activities and an annual bouldering competition offer people the opportunity to improve their skills and to socialize and connect with other climbers from all over Lebanon.

### Instruments

#### Primary outcome measure: mental well-being

Mental well-being was measured at both time points using the Levantine Arabic version [41] of the Warwick-Edinburgh Mental Well-Being Scale (WEMWBS). The Levantine version is a slightly modified version of the validated High Arabic version, with good psychometric properties, such as an internal consistency of 0.91 [42]. The WEMWBS consists of 14 items rated on a 5-point Likert scale covering emotional and functional aspects of mental well-being. Overall scores range from 14 to 70, with higher scores indicating greater overall mental well-being. In the European context, values below 42 are considered low mental well-being and values above 60 high mental well-being [43]. An improvement of 3 to 8 points is considered clinically relevant [44].

#### Secondary outcome measure

##### Psychological distress

Psychological distress was measured with the Arabic version of the Kessler psychological distress scale (K-6) [45, 46]. The Arabic version was validated in a Palestinian sample that speaks a very similar dialect to the one spoken in Syria or Lebanon. The K-6 consists of six items rated on a 5-point Likert scale ranging from 1 (*none of the time*) to 5 (*all of the time*), covering the main depressive symptoms (i.e., despair, feeling worthless, nervousness, so sad that nothing can cheer me up, and feeling that everything is an effort). Total scores ranged from 6 to 30, with higher scores signifying greater psychological distress. Factorial (*r* = 0.60, *p* < 0.001) and convergent validity (*r* > 0.60) with the Generalized Anxiety Disorder Scale- 7 (GAD-7) and Somatic Symptom-Scale 8 (SSS-8), respectively for the Arabic version was high. Internal consistency was high as well (Cronbach’s α = 0.81) [45]. The authors of the Arabic validation study used a score of 16.25 as the threshold for psychological disorders [45].

##### Self-efficacy

Self-efficacy was measured with the Arabic version of the General Self-Efficacy Scale (GSE) [47]. The 10 items are rated on a 4-point scale ranging from 1 (*not at all true*) to 4 (*exactly true*). The total score ranges from 10 to 40, with higher scores indicating higher levels of self-efficacy. The GSE has been shown to have good psychometric properties in intercultural samples [48] with an internal consistency of 0.95 in the Arab validation study. As the validation study was carried out in Qatar, where a different dialect is spoken, we checked for comprehension problems in a small sample of Syrian refugees. The original scale was not altered.

##### Social cohesion

Social cohesion was measured with 2 subscales from the Attitude, Reactions, and Knowledge (ARK) Regular Perception Survey [36]. This survey was used to assess social tension in Lebanon, particularly regarding the relationships between Syrian refugees and Lebanese host communities. The questions were modified to reflect the situation experienced by young people. The first subscale named ARK 1 contact in this paper referred to contact scenarios with members of the other nationality in play or attending school, living in close proximity, brought together through marriage, or sharing the same workplace. Each of the five items uses a 5-point Likert scale ranging from 1 (*very disagreeable*) to 5 (*very agreeable*) with a total score between 5 and 25. In the item named ARK2 values, participants were asked to indicate the extent to which they agreed with the sentence: "Lebanese and Syrians share many values and have compatible lifestyles" on a 4-point Likert scale ranging from 1 (*strongly agree*) to 4 (*strongly disagree*) [36]. The ARK is not designed to deliver a sum score, instead subscales or subitems are interpreted on its own.

#### Participants demographics

##### Physical activity

PA was measured with the Physical Activity Vital Sign (PAVS) [49]. PAVS scores are calculated by multiplying the reported days per week by average the minutes per day of at least moderate intensity exercise (e.g., walking, dancing). It consists of two questions: (a) How many days per week do you perform moderate to strenuous exercise (e.g., brisk walking, further examples given)? and (b) On average, how many minutes do you exercise per day? The PAVS showed good construct validity in the use with adults [50] and successfully detects common age-sex-BMI associations in a paediatric population [49].

The following variables were also assessed: sex, age, schooling, nationality, residency status, number of household family members, employment of family members, type of housing, and bouldering experience.

### Statistical analyses

#### Univariate analyses

Descriptive statistics (frequencies, means, and standard deviations) were computed to describe the sample and baseline characteristics. To assess the quality of the group allocation, differences between the IG and CG in the variables of interest were evaluated via two-sample *t* tests, U-tests, or chi-square (χ^2^) tests. The underlying assumptions of parametric tests were checked with the Shapiro–Wilk test and Levene’s test. If baseline variables differed significantly between the two groups, they were included as covariate in the multilevel analysis. All data were checked for plausibility. We conducted a missing data evaluation and made the assumption of data missing at random. There were no missing data on item level. Participants who attended fewer than four sessions (i.e., < 50% of the intervention) were subsequently interviewed and coded as dropouts. Dropout analyses were computed to check for differences between participants who dropped out and those who completed the study, using χ^2^ tests, U-tests, and two-sample *t* tests. For all outcomes, we report descriptive values before (pretest) and after the treatment (posttest). As a measure of effect size, Cohen’s *d* as the between-group effect size with adjusted means and the pooled standard deviation was calculated (IG adjusted mean minus CG adjusted mean) divided by the posttest (pooled unadjusted standard deviation) (calculated according to [51]).

#### Multilevel analysis

With the outcome variables (primary: mental well-being; secondary: psychological distress, self-efficacy, social cohesion) recorded twice for each participant (before and after the intervention), the data set had a hierarchical structure, with observations nested within participants treated as clusters. Therefore, the data were analyzed with Linear Mixed Models (LMMs), with a random intercept controlling for intracluster homogeneity. All available data points for all participants were included in the analyses (see Fig. 1), including dropouts in line with intention to treat analysis. Participants with missing time points were included in the LMMs under the assumption of MAR.

The respective outcome variable was modeled as a function of covariates in a series of models, starting from a *Null* model that contained only the fixed and random intercepts. Next, covariates were added, one at a time starting with group allocation, until the model fit was no longer improving. The hypotheses of research interest were tested with a Time Point (after vs. before) x Treatment Group (control and intervention) interaction in addition to the main effects and other predictors.

As a measure of within-cluster homogeneity, the Intra-Cluster Correlation (ICC) coefficient was calculated for the main outcome as the ratio of the variance of the random intercept and the total variance (i.e., the sum of the variance of the random intercept and the variance of the residual term). The modeling was performed with the nlme package in R (version 4.3.0). A *p*-value of *p* < 0.05 was considered significant.

## Results

### Description of study participants

After submitting the online application and getting invited to the trial sessions, 263 people attended the trial sessions and were screened. We were able to include 233 people in the program; of them, 156 (67%) fulfilled no dropout criteria (see Fig. 1). Posttests were available from 159 participants (68%). Participants were on average 16 years old, most of them were Syrian refugees (83%), and more than a half were female adolescents. There were no differences in age, sex, housing, residency status, or other baseline variables between IG and CG. Overall mental well-being was around 50 points, which can be considered average [43]. The level of distress was above the established cut-off of 14 points for the original [52] and 16.25 points for the Arabic version [45], indicating a high likelihood of psychological disorders [45]. No normative data existed for the Arabic groups on the level of self-efficacy. There were no significant differences on any variables between the completers and the individuals who dropped out (see Table 2). Cronbachs alpha in our sample ranged between α = 0.74 (K6) and α = 0.84 (ARK1).

**Table 2:** Sample characteristics (completers) and dropout analysis

Variable	IG (*n* = 83)	CG (*n* = 73)	Total (*n* = 156)	Test of group differences		Dropout (*n* = 77)	Test of group differences	
				χ^2^/T/U	p		χ^2^/T/U	p
Age^a^ *M* (*SD*)	16.12 (1.54)	15.97 (1.62)	16.05 (1.57)	− .52	.60	16.38 (1.53)	− 1.495	.136
Sex: female *n* (%)	51 (61.4)	40 (54.8)	91 (58.3)	.71	.40	35 (45.5)	3.44	.064
Enrolled in formal education yes *n*^*c*^ (%)	43 (51.8)	51 (69.9)	94 (60.2)	5.28	.174	40 (51.9)	1.46	.228
Minutes per week of physical activity^b^ MD(IQR)	120 (195)	105 (180)	120 (190)	2.96	.817	90 (155)	5288.0	.137
Residency status, refugee *n* (%)	72 (86.7)	58 (79.5)	130 (83.3)	1.49	.223	61 (79.2)	.590	.442
Kind of home: tent/container: yes, *n* (%)	34 (41.0)	26 (35.6)	60 (38.5)	.469	.493	33 (42.9)	.415	.519
Rent/own home rent, *n* (%)	72 (86.7)	58 (79.5)	130 (83.3)	1.49	.223	65 (84.4)	.044	.833
Previous experience with climbing: yes, *n* (%)	3 (3.6)	0 (0)	3 (1.9)	2.69	.101	3 (3.9)	.800	.371
Number of family members in household, *n* (%)				1.06	.304^e^		.011	.915^e^
Less than 5	8 (9.6)	6 (8.2)	14 (9.0)			5 (6.5)		
5	11 (13.3)	18 (24.7)	29 (18.6)			13 (16.9)		
6	14 (16.9)	11 (15.1)	25 (16.0)			15 (19.9)		
7	18 (21.7)	21 (28.8)	39 (25.0)			15 (19.9)		
8	17 (20.5)	6 (8.2)	23 (14.7)			12 (15.6)		
9 or more	15 (18.0)	11 (15.1)	26 (16.5)			17 (24.0)		
Employed household family members *n* (%)				.62	.431^d^		.2.65	.104^d^
0	11 (13.3)	13 (17.8)	24 (15.4)			6 (7.8)		
1	47 (56.6)	41 (56.2)	88 (56.4)			56 (59.7)		
2	21 (25.3)	16 (21.9)	37 (23.7)			15 (19.5)		
3–4	4 (4.8)	3 (4.1)	7 (4.4)			10 (13.0)		
Minutes of sport per week (Md)	120	105	120	2964.5	.817	90	5388.0	.173
Baseline measures (t0)								
WEMWBS, *M* (*SD*)^a^	50.49 (8.12)	49.93 (7.73)	50.23 (7.9)	− .44	.66	48.49 (9.08)	1.50	.135
K6, *M* (*SD*)^a^	17.20 (5.17)	16.64 (4.96)	16.94 (5.1)	− .69	.49	17.83 (5.00)	− 1.27	.207
GSE, *M* (*SD*)^a^	29.43 (5.10)	27.85 (5.75)	28.69 (5.5)	− 1.82	.07	28.12 (5.07)	.78	.439
ARK Q1, Md (IQR)^b^	9.0 (6.0)	8.0 (5.0)	8.4 (5.0)	3431.5	.45	8.0 (7.0)	6224.5	.645
ARK Q2, Md (IQR)^b^	2.0 (1.0)	2.0 (1.0)	2.0 (1.0)	2836.0	.14	2.0 (0.0)	6508.0	.248

*BMI* Body Mass Index; *WEMWBS* Warwick-Edinburgh Mental Well-Being Scale; *K-6* Kessler psychological distress scale; *GSE* General Self-Efficacy Scale

^a^The Shapiro–Wilk test indicated a significant deviation from a normal distribution in one of the subgroups, but no deviation was observed via histograms; therefore, the mean value (*M*) and standard deviation (*SD*) are reported

^b^Both the Kolmogorov–Smirnov test and an optical inspection (histogram) indicated a significant deviation from a normal distribution; therefore, the median (*MD*) and the interquartile range (*IQR*) are reported. A U-Test was used to compare group differences

^c^n = 124, information from 34 participants was unknown

^d^dichotomized: at least one family member employed vs. all unemployed

^e^dichotomized: 6 or fewer vs. 7 or more household members

Table 3 presents changes in descriptive values for all outcomes in the per protocol sample.

**Table 3:** Raw means and standard deviations at Pre and Post intervention and between-group mean differences and effect sizes calculated using pooled standard deviation and adjusted means (per protocol sample)

Variable	IG (*n* = 83)		CG (*n* = 73)		Between-group difference, IG-CT	Between-group effect size^§^
	Pre *M* (*SD*)	Post *M* (*SD*)	Pre M (SD)	Post *M* (*SD*)	*M* (95% CI)	|*d*|
WEMWBS	50.5 (8.12)	53.6 (8.1)	49.9 (7.7)	49.1 (7.7)	− 3.94 (− 6.29–− 1.60)	.45
K6	17.2 (5.17)	14.87 (5.48)	16.64 (4.96)	17.14 (4.98)	2.83 (1.21–4.45)	.61
GSE	29.43 (5.10)	30.31 (4.91)	27.85 (5.75)	27.75 (4.81)	− .98 (− 2.52–.57)	.06
ARK contact	8.95 (3.91)	8.31 (3.84)	7.95 (3.15)	8.25 (3.50)	.94 (− .07–1.95)	.08
ARK values	1.93 (.87)	1.81 (.63)	2.00 (.80)	1.74 (.55)	− .14 (− .40–.12)	.17

*IG* intervention group; *CG* control group; *WEMWBS* Warwick Edinburgh Mental Wellbeing Scale, *K-6* Kessler psychological distress scale; *GSE* General Self-Efficacy Scale; *PP* per protocol; *Post* Posttest; *Pre* Pretest

^§^Cohen’s *d* based on the pooled standard deviation and adjusted means, WEMWBS, K-6, and GSE were in favor of the IG; ARK was in favor of the CG

### Main outcome: overall mental well-being

After the *Null* model and the second model, which accounted for the two time points, we included the IG (reference: CG) and its interaction with the timepoints as the most important effect from a research point of view in Model 3 (see Table 4).

**Table 4:** Overview of models for overall mental well-being

Model	Specification	Fit	
Model 1	Design variables (outcome and patient number)	AIC	2720.298
		BIC	2732.212
		logLik	− 1357.149
Model 2	Model 1 + time	AIC	2715.864
		BIC	2731.749
		logLik	− 1353.932
Model 3	Model 2 + group and group x Time	AIC	2705.062
		BIC	2728.89
		logLik	− 1346.531
Model 4	Model 3 + cycle	AIC	2698.34
		BIC	2726.139
		logLik	− 1342.17
Model 5	Model 4 + schooling	AIC	2697.923
		BIC	2729.693
		logLik	− 1340.961
Model 6	Model 5 + housing	AIC	2691.83
		BIC	2727.571
		logLik	− 1336.915
Model 7	Model 6 + dropout	AIC	2689.962
		BIC	2729.675
		logLik	− 1334.981

*AIC* Akaike information criterion; *BIC* Bayesian information criterion; *logLik* log Likelihood

**Fig. 2:**
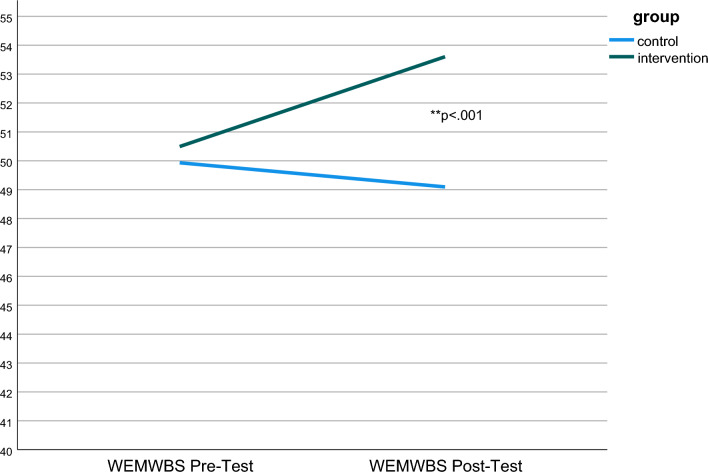
Differences in the WEMWBS pretest and posttest between the groups. Green line: intervention group/blue line: control group

We then successively included predictors and tested the goodness-of-fit indices. A few siblings attended the same intervention group (n = 5), which could have led to the inclusion of an additional level ‘family’. However, this level would have been extremely unbalanced, as most of them would have consisted of a single subject and therefore would not have contributed to the intra-cluster correlation. We therefor tested the following predictors: age, sex, dropout, type of house, enrolled in formal education, residency status, minutes of sports per week, family employment. Model 7 showed the best fit and is presented as the final model (random effect: Var 5.91 (Intercept), 5.21 (residual); ICC 0.56). In all models, the interaction between group and time was highly significant (Table 5), indicating a direct intervention effect of the YouCLIMB intervention on mental well-being. Members of the IG improved by approximately 4 points during the intervention, which can be considered clinically relevant [44]. The within group effect size was *d* = 0.43 95% CI [0.13, 0.74] (r = 0.56) (Fig. 2).

**Table 5:** Fixed effect estimates from the best-fitting models for overall psychological well-being (coefficients and standard errors)

Predictor	Model 5			Model 6			Model 7		
	Coeff.	SE	*p*	Coeff.	SE	*p*	Coeff.	SE	*p*
Constant	52.152	1.061		50.624	1.175		51.678	1.288	
Time	− 0.668	0.834	.424	− 0.656	0.834	.432	− 1.025	0.854	.232
Cycle 2 (ref. cycle 1)	− 2.890	0.991	**.004**	− 2.386	0.992	**.017**	− 2.530	0.989	**.011**
IG (ref. CG)	− 0.047	1.073	0.97	− 0.017	1.059	.987	− 0.312	1.065	.770
In formal education (ref. not enrolled)	− 1.539	0.995	0.12	− 2.533	1.041	**.016**	− 2.650	1.036	**.011**
Housing				3.009	1.059	**.005**	2.936	1.053	**.006**
Dropout							− 2.184	1.119	.052
Group × time interaction	4.113	1.143	** < .001**	4.082	1.143	** < .001**	4.242	1.145	** < .001**

Significant *p*-values are marked in bold

*Coeff* coefficient; *SE* standard error

Other predictors also had significant *p*-values. Participants who lived in a nonpermanent home and were still enrolled in formal education had lower scores than those who lived in a permanent home and were not in formal education. Interestingly, cycle was also a significant variable, with people in the first cycle having higher scores at both the first and last measurement points, regardless of group. We therefore tested for a Cycle x Time interaction in a separate model, which was far from significant (*p* = 0.96). The two cycles had completely parallel slopes, but overall well-being in Cycle 1 began 2 points higher.

### Secondary outcomes

For the multilevel analysis, the same statistical procedure was applied as for the primary outcome. Table 6 gives an overview of the results for the secondary outcomes. The included predictors reflect the best fitting model.

**Table 6:** Fixed effect estimates from the best fitting models for secondary outcomes (coefficients and standard errors)

Predictor	Final model K-6			Final model GSE			Final model ARK1			Final model ARK2		
	Coeff.	SE	*p*	Coeff.	SE	*p*	Coeff.	SE	*p*	Coeff.	SE	*p*
Time	0.391	0.565	.491	− 0.301	0.558	.591	0.405	0.371	.276	− 0.278	0.090	**.003**
IG (ref. CG)	0.723	0.670	.282	0.749	0.672	.266	1.265	0.483	**.009**	− 0.094	0.094	.316
Group × time interaction	− 3.009	0.776	** < .001**	0.266	0.748	.071	− 1.036	0.498	**.039**	0.132	0.125	.293
Residency	1.557	0.788	**.049**									
Sex				− 1.005	0.628	.111	− 0.817	0.458	.076			
Housing				1.252	0.670	.063	− 0.330	0.490	.501	.157	0.090	.085
Dropout				− 0.817	0.705	.248	0.344	0.508	.499			
In formal education (ref. not enrolled)				− 1.591	0.650	**.015**	− 1.148	0.475	**.016**	− 0.161	0.088	.069
Cycle 2 (ref. cycle 1)				− 1.4418	0.621	**.021**	0.094	0.453	.837	0.154	0.083	.067
Age										− 0.051	0.027	.060

Significant *p*-values are printed in bold

*Ref.* reference

Being a refugee significantly increased the amount of psychological distress, whereas participating in the IG led to a significant reduction in psychological distress.

For self-efficacy, being enrolled in formal education and participating in the second cycle predicted lower self-efficacy. Participating in the intervention group sharply missed the significance threshold for predicting an improvement in self-efficacy.

An unexpected outcome was shown for social cohesion: For the contact item, the groups were already significantly different at baseline, with the CG showing lower values, but during the intervention, the two groups’ values approached each other, resulting in significant values for group and the interaction of group and time. Additionally, being enrolled in formal education led to lower social cohesion scores with respect to acceptability of contact with the other nationality.

For the second social cohesion item (shared values), there was only a main effect of time with the control and IGs both agreeing more with the idea of shared values with time. Differences in social cohesion relied on only one item each, and differences were very small (see Table 6 for an overview of the secondary outcomes).

### Adverse events

During the intervention period, no severe adverse advents were reported. One moderate adverse event occurred in the IG with a participant suffering a fractured ankle that required treatment at the local hospital. Costs were partly covered by United Nations High Commissioner for Refugees (UNHCR) and partly covered by ClimbAID. By the time of submission, the participant had fully recovered. Another participant suffered a fractured leg in a car accident during the study period, but it was unrelated to the intervention.

## Discussion

This study reports evidence that an MHPSS-informed bouldering intervention significantly improved mental well-being compared with a control group (between group effect size from adjusted means *d* = 0.45) in a sample of young refugees and members of the host community in the Bekaa region of Lebanon. Our findings make an important contribution to the increasing literature on the mental health benefits of sports-based interventions in humanitarian and displacement contexts [23, 53–55]. The findings confirmed our primary hypothesis that the intervention would improve mental well-being, as we found a clinically meaningful change in mental well-being [44, 56]. Significant differences between the intervention and control groups were also found for psychological distress (effect size from adjusted means *d* = 0.60). The results are in line with previous literature from both high- and low-resource contexts [9, 56–58]. No impact of the intervention was found for self-efficacy or social cohesion.

The intervention consisted of an 8-week community-based MHPSS-informed bouldering program, designed to foster mental well-being and reduce psychological distress at a preventative level. A theory of change for therapeutic bouldering includes mindfulness, emotional intensity, a large probability of transfer, learning in and with the group, and quick improvements in beginners [59].

Positive effects of PA in humanitarian or forced displacement settings were recently validated in a meta-analysis for a number of outcomes including psychological symptoms, self-efficacy, and coping with a trend for mental well-being [23]. However, some studies have also had unintended outcomes [60]. For example, a sport for development program in Uganda showed that competitive sports can have a negative impact on mental health outcomes [18]. Besides the differences in the type of sport (competitive football vs. cooperative bouldering) and sample (child soldiers vs. refugees), another possible explanation for the differences between the results of the study by Richards et al. and the current project is the fact that coaches in the Uganda evaluation had not been given specific training in mental health nor did they have access to supervision and support from a mental health practitioner. In a recent feasibility study on refugee youth in Lebanon, intensified training for the facilitators was also one of the modifications found to be necessary after the pilot study [61]. A recent publication by the Olympic Refugee Foundation [62] also argued for the importance of integrated training in foundational skills, such as psychological first aid (PFA).

Most of our results are in line with the recent meta-analysis [23], with significant improvements in metal well-being and psychological distress, but we found that participation in the IG did not quite reach statistical significance in predicting higher self-efficacy values. Unfortunately, normative data are not available for an Arab population. However, the mean of 28.69 is comparable to the mean of a large German norm sample [63]. This comparability may indicate that this value could also be considered average in our sample, which would be the desired and healthy outcome, thereby not leaving enough room for improvement. Another reason could be that the scale for self-efficacy has been validated in a rather different sample from ours, and should culturally and contextually be validated in Syrian and Lebanese adolescents.

Unexpected results were found for one of the two social cohesion items. Because the host country participants and refugees participated in YouCLIMB together, we hypothesized that having this shared experience would lead to a positive impact on social cohesion and integration between the host country participants and the refugees. The shared experience of climbing and other activities could foster a sense of trust, respect, and mutual support among participants, which could help participants overcome social barriers and prejudices. However, the YouCLIMB curriculum only indirectly addresses social cohesion via cooperation and trust, which might have led to the lack of effect on social cohesion. Additionally, we had a heterogenous sample, and despite the program being open to all nationalities, most of the participants were Syrian, and only around 17% were Lebanese (compared with around 60% in the population in the Bekaa [64]). This proportion might simply have been too low to foster enough contact between ethnicities. The integration of more Lebanese adolescents was also hindered by the fact that separation between Syrian and Lebanese youth is part of the current political and educational landscape. For example, the separation of Syrian and Lebanese children throughout Lebanon is currently ensured through policies, such as different schooling hours in many schools, which has hindered social interaction and contributed to mutual distrust between these communities [65]. Future studies are required to verify the findings because differences between the groups in social cohesion were very small. Furthermore, the conceptualization of social cohesion relied on only one item each and should be reviewed in future studies [66].

Our study had a number of strengths, including the controlled design. In a recent meta-analysis, one third of the included studies had no control group [23], which has also been the case for MHPSS support in interventions [9]. Our sample size exceeded the size calculated as necessary in the power calculation, and was larger than the vast majority of studies on child and adolescent mental health and psychosocial support in the Middle East, which have operated with a sample size of 60 or less. [7]. Dropout rates in our study were around 35%, which is comparable to other studies [67] in the same environment, for example, the Advancing Adolescence study [56]. The treatment attendance rate of 75% can be considered a good completion rate obtained in a challenging, real-world environment [56, 61]. Nevertheless attrition rates need to be explored further in order to determine ways to retain participants in the intervention. Effect sizes for mental well-being and psychological distress were rather high for the complex environment and can be regarded as clinically significant [56]. We also achieved our goal of including a large proportion of female participants in the sample, as a lack of female participants has widely been identified as one of the main limitations of existing literature in this area [68]. In a region where traditional gender stereotypes often hinder the participation of women in sports activities, ClimbAID has made it a priority to promote the inclusion of women. To achieve equal participation of women, for several years, ClimbAID has therefore been investing in training female participants so that they can become session facilitators and can serve as role models for female intervention participants. Climbing is not recognized as a culturally traditional sport in the region which may have had the advantage of not being associated with particular gender stereotypes often associated with other sports [69].

Our results should be interpreted in light of limitations. First, this study was conducted in a complex humanitarian setting in which a number of external factors had to be considered and accounted for. Randomization of the entire sample was not possible due to cultural and organizational considerations. Future studies should involve fully powered RCTs to allow conclusive testing of causal effects. Also blinding was not possible because the results relied on self-reports. We searched for validated questionnaires and were able to include questionnaires that were at least validated in Arabic and often in Levantine Arabic. But nonetheless, most questionnaires, including the primary outcome, were validated only for adults and not for youth. We included youth between 14 and 19 years of age, and it can be assumed that their understanding of the items might be similar to the understanding of adults, but surely, there is a need for validated instruments for youth in Levantine Arabic. Also due to financial, organizational, and political reasons, we were not able to perform the intended follow-up. Effect sizes refer to the sample of completers which might lead to overestimations of the effect regarding the ITT-sample.

Other than in 2017 when ClimbAID started its activities and even in 2022 when the study took place, the Lebanon of 2024 is a much harder environment in which to implement community-based MHPSS-informed bouldering activities, as the needs of the population have shifted toward more basic needs, such as nutrition, shelter, and protection. Outreach, transportation, and group-session scheduling require many resources, especially in regions of protracted crises where transportation is costly or unsafe, and dropout is likely due to recurring life events. These important points were also mentioned in a recent feasibility study, WarChild conducted from September 2018 to July 2019 in Northern Lebanon [61]. Ultimately, the particular advantages offered by a climbing-related intervention must be balanced with the available resources in each context in order to achieve optimum effectiveness and impact. In future studies the inclusion of children with disabilities should further be explored, which is actually done in a specific curriculum by ClimbAID.

## Conclusions

This study is the first to demonstrate that a psychosocial bouldering intervention, conducted in the context of forced displacement, can significantly improve psychological well-being and reduce psychological distress in adolescents. The study found that bouldering is an effective and safe sport in the context of forced displacement in Lebanon, a lower middle income country. As physical inactivity is a risk factor not only for poor physical health but also for poor mental health, bouldering may therefore be a promising, novel target for interventions with adolescents in humanitarian settings [23, 40, 70, 71]. The results have important implications for the design and delivery of scalable psychosocial interventions and the role of sports-based (bouldering) approaches, including the recognition of sports-based interventions as integrated components of MHPSS programming.

## Supplementary Information

Supplementary Material 1.

## Data Availability

The data sets used and/or analyzed during the current study are available from the corresponding author upon reasonable request.

## Abbreviations

ARK: Attitude, reactions and knowledge
BMI: Body mass index
CEUA: Ethics Committee of Antonine University
CG: Control group
GAD-7: Generalized Anxiety Disorder-7
GSE: General Self-Efficacy Scale
IASC: Interagency Standing Committee
ICC: Intra-Cluster Correlation
IG: Intervention group
IOM: International Agency for Migrations
K-6: Kessler psychosocial distress scale
LMICs: Low- and middle-income countries
LMMs: Linear Mixed Models
MHPSS: Mental health and psychosocial support
PA: Physical activity
PAVS: Physical Activity Vital Sign
PTSD: Posttraumatic stress disorder
SSS-8: Somatic Symptom Scale-8
UNHCR: United Nations High Commissioner for Refugees
UNICEF: United Nations Children’s Emergency Fund
WEMWBS: Warwick-Edinburgh Mental Well-Being Scale
