# Supplementary material for: A psychosocial bouldering intervention improves the well-being of young refugees and adolescents from the host community in Lebanon: results from a pragmatic controlled trial
Source: Confl Health. 2024 Sep 14;18:56. doi: 10.1186/s13031-024-00615-3 (PMC11402205; doi:10.1186/s13031-024-00615-3)
Supplement: Supplementary file 1 — Supplementary Material 1. [file 13031_2024_615_MOESM1_ESM.docx]

Table 1. CONSORT 2010 checklist of information to include when reporting a clinical trial (adapted from <http://www.consort-statement.org/>).

| Section/Topic | Item No | Checklist item | Reported on page No |
| --- | --- | --- | --- |
| Title and abstract | | | |
|  | 1a | Identification as a randomised trial in the title | n.a. |
|  | 1b | Structured summary of trial design, methods, results, and conclusions (for specific guidance see CONSORT for abstracts) | Abstract |
| Introduction | | | |
| Background and objectives | 2a | Scientific background and explanation of rationale | 3ff |
|  | 2b  ext | Specific objectives or hypotheses  Describe the health or health service problem that the intervention is intended to address, and other interventions that may commonly be aimed at this problem. | 3ff |
|  |  |  | 3ff |
| Methods | | | |
| Trial design | 3a | Description of trial design (such as parallel, factorial) including allocation ratio | 6f |
|  | 3b | Important changes to methods after trial commencement (such as eligibility criteria), with reasons | n.a. |
| Participants | 4a | Eligibility criteria for participants | 7f |
|  | 4b  Ext. | Settings and locations where the data were collected  Eligibility criteria should be explicitly framed to show the degree to which they include typical participants and, where applicable, typical providers (eg, nurses), institutions (eg, hospitals), communities (or localities eg, towns) and settings of care (eg, different healthcare financing systems). | 9 |
|  |  |  | 7f |
| Interventions | 5  Ext. | The interventions for each group with sufficient details to allow replication, including how and when they were actually administered  Describe extra resources added to (or resources removed from) usual settings in order to implement the intervention. Indicate if efforts were made to standardise the intervention or if the intervention and its delivery were allowed to vary between participants, practitioners or study sites. Describe the comparator in similar detail to the intervention. | 11f |
|  |  |  | 11f |
| Outcomes | 6a | Completely defined pre-specified primary and secondary outcome measures, including how and when they were assessed | 13ff and 9 |
|  | 6b  Ext. | Any changes to trial outcomes after the trial commenced, with reasons  Explain why the chosen outcomes and, when relevant, the length of follow-up are considered important to those who will use the results of the trial | n.a. |
|  |  |  | 3f /n.a. |
| Sample size | 7a | How sample size was determined | 9 |
|  | 7b  ext | When applicable, explanation of any interim analyses and stopping guidelines  If calculated using the smallest difference considered important by the target decision maker audience (the minimally important difference) then report where this difference was obtained. | 10 |
|  |  |  | n.a. |
| Randomisation: |  |  |  |
| Sequence generation | 8a | Method used to generate the random allocation sequence | 8f |
|  | 8b | Type of randomisation; details of any restriction (such as blocking and block size) | 8f |
| Allocation concealment mechanism | 9 | Mechanism used to implement the random allocation sequence (such as sequentially numbered containers), describing any steps taken to conceal the sequence until interventions were assigned | 8f |
| Implementation | 10 | Who generated the random allocation sequence, who enrolled participants, and who assigned participants to interventions | 8f |
| Blinding | 11a | If done, who was blinded after assignment to interventions (for example, participants, care providers, those assessing outcomes) and how | 9 |
|  | 11b  Ext. | If relevant, description of the similarity of interventions  If blinding was not done, or was not possible, explain why | n.a |
|  |  |  | 9 |
| Statistical methods | 12a | Statistical methods used to compare groups for primary and secondary outcomes | 15f |
|  | 12b | Methods for additional analyses, such as subgroup analyses and adjusted analyses | n.a. |
| Results | | | |
| Participant flow (a diagram is strongly recommended) | 13a | For each group, the numbers of participants who were randomly assigned, received intended treatment, and were analysed for the primary outcome | Fig. 1 |
|  | 13b  Ext. pt | For each group, losses and exclusions after randomisation, together with reasons The number of participants or units approached to take part in the trial, the number which were eligible and reasons for non-participation should be reported. | Fig 1  Fig 1 |
| Recruitment | 14a | Dates defining the periods of recruitment and follow-up | 8 |
|  | 14b | Why the trial ended or was stopped | n.a. |
| Baseline data | 15 | A table showing baseline demographic and clinical characteristics for each group | Table 2 |
| Numbers analysed | 16 | For each group, number of participants (denominator) included in each analysis and whether the analysis was by original assigned groups | 17f |
| Outcomes and estimation | 17a | For each primary and secondary outcome, results for each group, and the estimated effect size and its precision (such as 95% confidence interval) | Table 3 |
|  | 17b | For binary outcomes, presentation of both absolute and relative effect sizes is recommended | n.a. |
| Ancillary analyses | 18 | Results of any other analyses performed, including subgroup analyses and adjusted analyses, distinguishing pre-specified from exploratory | 22 |
| Harms | 19 | All important harms or unintended effects in each group (for specific guidance see CONSORT for harms) | 24 |
| Discussion | | | |
| Limitations | 20 | Trial limitations, addressing sources of potential bias, imprecision, and, if relevant, multiplicity of analyses | 27 |
| Generalisability | 21  Ext.pt | Generalisability (external validity, applicability) of the trial findings;  Describe key aspects of the setting which determined the trial results. Discuss possible differences in other settings where clinical traditions, health service organisation, staffing, or resources may vary from those of the trial. | 24ff |
|  |  |  | 24ff |
| Interpretation | 22 | Interpretation consistent with results, balancing benefits and harms, and considering other relevant evidence | 24ff |
| Other information | | |  |
| Registration | 23 | Registration number and name of trial registry | 2 |
| Protocol | 24 | Where the full trial protocol can be accessed, if available | 6 |
| Funding | 25 | Sources of funding and other support (such as supply of drugs), role of funders | 31 |
